# Effect of Sodium Selenite on the Metabolite Profile of *Epichloë* sp. Mycelia from *Festuca sinensis* in Solid Culture

**DOI:** 10.1007/s12011-021-03054-w

**Published:** 2022-01-01

**Authors:** Lianyu Zhou, Lu Jiao, Jiasheng Ju, Xuelan Ma

**Affiliations:** Key Laboratory of Medicinal Plant and Animal Resources of the Qinghai-Tibetan Plateau, Academy of Plateau Science and Sustainability, School of Life Science, Qinghai Normal University, Xining, 810008 China

**Keywords:** *Epichloë* sp. from *Festuca sinensis*, Selenium, Culture time, Metabolite analysis

## Abstract

Selenium (Se) is an essential micronutrient with many beneficial effects for humans and other living organisms. Numerous microorganisms in culture systems enrich and convert inorganic selenium to organic selenium. In this study, *Epichloë* sp. from *Festuca sinensis* was exposed to increasing Na_2_SeO_3_ concentrations (0, 0.1, 0.2, 0.3, and 0.4 mmol/L) in Petri dishes with potato dextrose agar (PDA) for 8 weeks. *Epichloë* sp. mycelia were immediately collected after mycelial diameters were measured at 4, 5, 6, 7, and 8 weeks of cultivation, respectively. Gas chromatography-mass spectrometer (GC-MS) analysis was performed on different groups of *Epichloë* sp. mycelia. Different changes were observed as *Epichloë* sp. was exposed to different selenite conditions and cultivation time. The colony diameter of *Epichloë* sp. decreased in response to increased selenite concentrations, whereas the inhibitory effects diminished over time. Seventy-two of the 203 identified metabolites did not differ significantly across selenite treatments within the same time point, while 82 compounds did not differ significantly between multiple time points of the same Se concentration. However, the relative levels of 122 metabolites increased the most under selenite conditions. Specifically, between the 4th and 8th weeks, there were increases in 2-keto-isovaleric acid, uridine, and maltose in selenite treatments compared to controls. Selenium increased glutathione levels and exhibited antioxidant properties in weeks 4, 5, and 7. Additionally, we observed that different doses of selenite could promote the production of carbohydrates such as isomaltose, cellobiose, and sucrose; fatty acids such as palmitoleic acid, palmitic acid, and stearic acid; and amino acids such as lysine and tyrosine in *Epichloë* sp. mycelia. Therefore, *Epichloë* sp. exposed to selenite stress may benefit from increased levels of some metabolite compounds.

**Supplementary Information:**

The online version contains supplementary material available at 10.1007/s12011-021-03054-w.

## Introduction

Selenium (Se) is a vital micronutrient for animals and is thought to have anticancer, immune-enhancing, and antioxidant properties [1]. The majority of microorganisms are capable of bioaccumulating selenium in its inorganic forms such as selenite and selenate and convert to organic forms such as selenoproteins, Se-lipids, Se-peptides, and Se-amino acids [2, 3]. It is worth noting that different concentrations of Se can affect the growth and metabolic processes in microorganisms. Currently available scientific research on selenium’s effect on fungal properties are focused on growth [2], morphology [4, 5], antioxidation [6–8], selenium metabolomics [3, 9], and chemical compounds [6, 10, 11]. In general, selenium tolerance is often associated with the accumulation of organic Se in certain fungi [12]. Previously, the selenium-tolerance mechanism of microorganisms has been investigated using genomic [13], proteomics [14], and metabolomic analysis [11]. Metabolomics has a great potential to significantly advance our understanding of how microorganisms survive elemental stress through the use of small molecules [15].

Plant endophytes grow biotrophically in host grasses in nature, but nearly all may be grown saprotrophically in culture using potato dextrose agar (PDA) or nutrient agar (NA). They can help plants cope with stress by promoting growth and regulating selenium accumulation [12, 16]. The asexual *Epichloë* endophytes are formally referred to as *Neotyphodium* spp. may confer benefits on economically significant forage grasses, including resistance to biotic and abiotic stressors [17, 18]. Numerous research has been conducted to elucidate the biological and physiological properties of *Epichloë* endophytes [19, 20], while the ability of *Epichloë* species to withstand stress has been associated with host adaptability [21].

*Festuca sinensis* is an economically important cool-season grass species widely distributed in the cool and semi-arid regions of China, and it is grazed by cattle and sheep. This grass species is frequently hosted to an asexual symptomless *Epichloë* sp. which has been shown to improve the response of *F*. *sinensis* to stressors such as drought, waterlogging, cold, and pathogens [22–25], and to produce alkaloids in the host tissues [26, 27]. The morphological, physiological, and bioactivity properties of *Epichloë* sp. from *F*. *sinensis* have been described as being diverse [19, 25, 28]. Due to their morphological and phylogenetic characteristics, the endophyte strains found in *F*. *sinensis* have been renamed *E. sinensis* [29]. Xu et al. [16] reported that a high concentration of Na_2_SeO_4_ inhibits endophytic bacterial growth and that the endophytic *Herbaspirillum* sp. cultured in LB broth containing 200 mmol/L Na_2_SeO_4_ reduced selenate to elemental selenium. However, information on the mechanisms of action of selenite on *Epichloë* sp. is scarce.

The current study used a metabolomics approach to deduce the metabolic changes that occurred in *Epichloë* sp. subjected to different selenite concentrations for 8 weeks. Studies on the metabolic mechanisms by which endophytes metabolize selenium are critical in the case of symbiosis associated with selenite adaption.

## Materials and Methods

### Epichloë sp. Strain

*Epichloë* endophyte strain obtained from Key Laboratory of Medicinal Plant and Animal Resources of the Qinghai-Tibetan Plateau, Qinghai Normal University, was grown on potato dextrose agar media (200 g potato infusion, 20 g dextrose, 20 g agar, 1000 mL distilled water) [25]. Inoculation colonies were prepared using 30-day cultures of *Epichloë* sp. on PDA media.

### Effects of Se on the Growth and Metabolites of Epichloë sp.

To analyze *Epichloë* sp. tolerance to different selenite concentrations, three 4-mm diameter mycelial agar plugs were placed in a triangle in a sealed Petri dish containing PDA medium supplemented with Na_2_SeO_3_ at five concentrations 0 (CK), 0.1, 0.2, 0.3, or 0.4 mmol/L, respectively, following a pre-test with 0, 1.0, 2.0, 3.0, or 4.0 mmol/L Se, respectively. All cultures were incubated for 4~8 weeks at a constant temperature of 25±1℃. The fungal growth rate was determined by measuring the diameter of each colony once a week from the 4th to 8th week. The experimental design included eight replicates for each treatment, with three mycelial agar plugs on each plate.

Mycelia (20 mg) from each treatment were harvested in the 4th, 5th, 6th, 7th, and 8th weeks, respectively, by scraping off the surface of multiple colonies and immediately quenched in 200 μL methanol (–20℃), and stored at –80℃ for determination of metabolites.

### Metabolite Extraction and Derivatization

A total of 20 mg of mycelial material were extracted using 1.0 mL of extraction mix (*V*_Methanol_:*V*_Chloroform_ = 3:1, volume ratio) and 5 μL of L-2-chlorophenylalanine (1 mg/mL stock in dH_2_O) as an internal standard. The mixture was homogenized for 4 min at 45 Hz and then treated with ultrasound for 5 min in an ice bath. This process was repeated three times, and the extract was then centrifuged at 4℃ for 15 min at 12000 rpm. Eight hundred microliters of the supernatant was carefully transferred into a new 1.5-mL EP tube, and 120 μL was pooled from each sample as a QC sample and dried in a vacuum concentrator without heating.

The pellet was resuspended in 30 μL of methoxyamination hydrochloride (20 mg/mL in pyridine) and incubated at 80℃ for 30 min. Subsequently, 40 μL of the BSTFA regent (1% TMCS, v/v) was added to the sample aliquots and incubated at 70℃ for 1.5 h, followed by the addition of 5 μL FAMEs (in chloroform) to the QC sample upon cooling to room temperature. Analysis of mycelial metabolites was carried out in triplicates.

### GC-TOF-MS Analysis

Samples were analyzed using an Agilent 7890 gas chromatograph system coupled with a Pegasus HT time-of-flight mass spectrometer and a DB-5MS capillary column coated with 5% diphenyl cross-linked with 95% dimethylpolysiloxane (30-m×250-μm inner diameter, 0.25-μm film thickness, J&W Scientific, Folsom, CA, USA). A 1-μL aliquot of sample was injected in splitless mode. The initial temperature was maintained at 50℃ for 1 min, then increased to 310℃ at a rate of 10℃/min for 8 min. Helium was used as the carrier gas at a rate of 1 mL/min. The injection, transfer line, and ion source temperatures were maintained at 280℃, 280℃, and 250℃, respectively. An electron impact of 70 eV was used for the ionization. After a solvent delay of 6.17 min, mass spectra were obtained in the *m/z* range of 50 to 500 at 12.5 scans/s.

### Data Processing and Statistical Analysis

Chroma TOF 4.3X software from LECO Corporation and LECO-Fiehn Rtx5 database was used to extract raw peaks, filter the data against a baseline, calibrate the baseline, align the peaks, perform deconvolution analysis, identify the peaks, and integrate the peak area. The mass spectrum and retention index were used to confirm the identification of metabolites. Additionally, peaks detected in <50% of QC samples or with an RSD>30% in QC samples were deleted.

For each metabolite examined, relative quantification was estimated using an internal standard. SPSS software, Version 16.0 (SPSS, Inc., Chicago, IL, USA) was used to perform principal component analysis (PCA). To determine the degree of metabolite abundance changes, MeV software was used to construct a heatmap and hierarchical clustering. Pathway maps and time courses of metabolite abundances were constructed in VANTED. The average diameter of colonies and metabolite data were determined, as well as the standard deviation of the means (SD). Using SPSS 16.0 software, data were compared using repeated measurement of one-way analysis of variance and least significant difference (LSD) at a significance level of *P*<0.05.

## Results

### Effect of Selenite Concentration on the Colony Diameter of Epichloë sp. from F. sinensis

**Figure 1:**
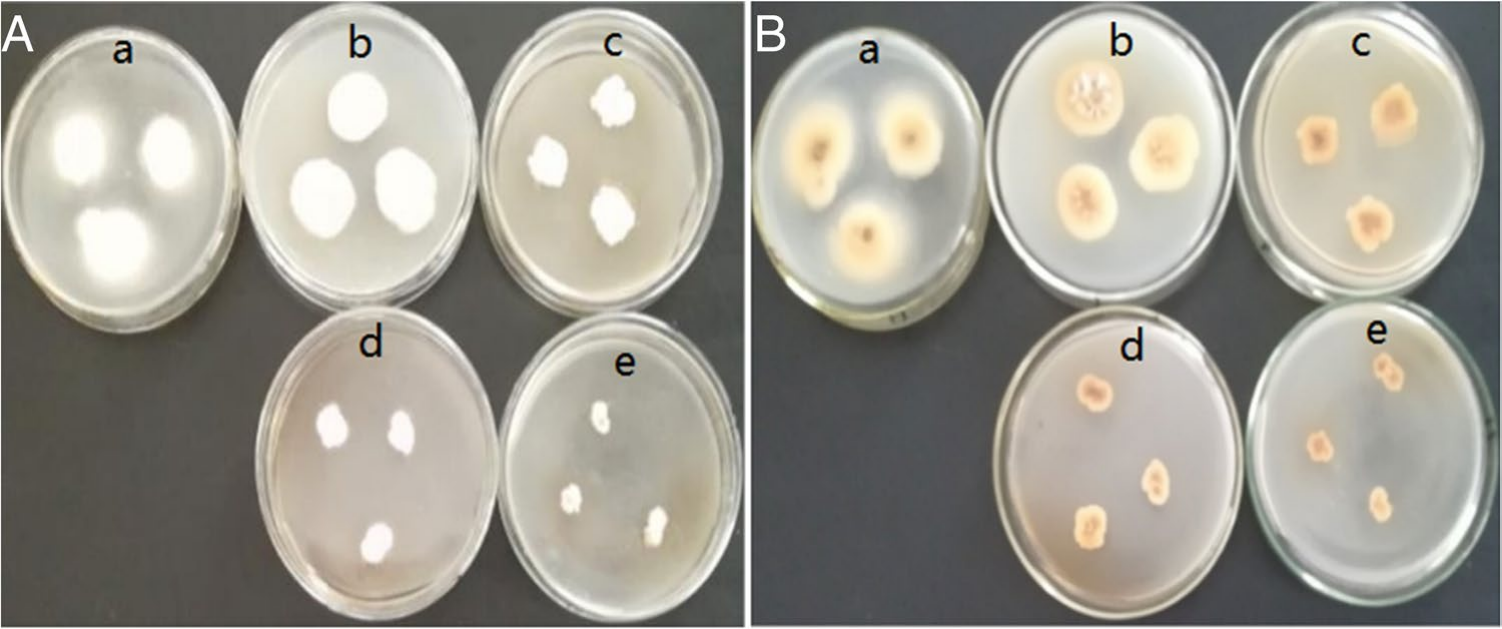
Effects of selenium supplementation on colony diameter of *Epichloë* sp. from *F. sinensis* at the eighth week. Obverse (A) and reverse (B) view of *Epichloë* sp. The five Petri dishes (a, b, c, d, e) were added at final selenium concentrations of 0, 0.1, 0.2, 0.3, or 0.4 mmol/L, respectively

As shown in Table 1 and Fig. 1, selenite inhibited the mycelial growth of *Epichloë* sp. from *F. sinensis* on PDA*.* After 4~6 weeks of cultivation, selenite concentrations ranging from 0.1 to 0.4 mmol/L significantly decreased mycelial growth (*P*<0.05). In comparison to the control concentration, the colony diameters generated by 0.1 mmol/L and 0.2 mmol/L Se did not show any significant change as the incubation time increased. However, the colony diameters inhibited by 0.3 and 0.4 mmol/L Se were significantly smaller than those in the control group (0 mmol/L) (*P*<0.05).

**Table 1:** Colony diameter of *Epichloë* sp. from *F. sinensis* cultivated in media containing different Se concentrations

Selenium (mmol/L)	Colony diameter(mm)				
	Week 4	Week 5	Week 6	Week 7	Week 8
0	19.09±0.37^dA^	22.67±0.33^cA^	24.51±0.30^bA^	30.78±0.42^aA^	30.16±0.32^aA^
0.1	15.39±0.29^eB^	19.59±0.32^dB^	21.64±0.23^cB^	26.40±0.48^bAB^	29.42±0.56^aA^
0.2	12.80±0.22^eC^	17.16±0.28^dC^	21.58±0.30^cB^	23.15±0.55^bB^	27.97±0.39^aAB^
0.3	10.32±0.34^eD^	13.97±0.37^dD^	17.88±0.51^cC^	21.64±0.23^bB^	24.08±0.42^aB^
0.4	7.38±0.39^cE^	10.07±0.49^bE^	11.95±0.55^bD^	19.22±0.64^aC^	19.34±0.52^aB^

Note: Data represent means ± standard deviation (SD). Means followed by different lowercase letters differed statistically under the same selenium concentration (*P*<0.05). Means followed by different uppercase letters differed statistically among a given culture time(*P*<0.05)

### Identification of Metabolites and Screening for Differential Metabolites

A of total 676 peaks and about 203 identifiable metabolites were detected in samples by mass spectrum and retention index matching, while 141 peaks and 268 metabolites remained unidentified in the database.

### Principal Component Analysis

**Figure 2:**
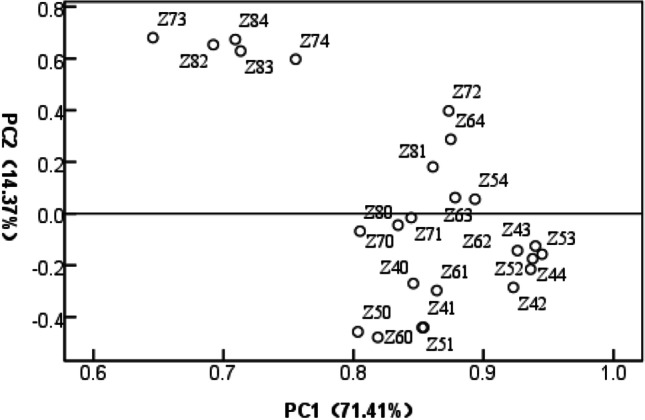
Principal component analysis (PCA) score plot of first and second PCs from 25 mycelial samples. Z40, Z41, Z42, Z43, and Z44 represented mycelia grown for 4 weeks in the presence of 0, 0.1, 0.2, 0.3, or 0.4 mmol/L Na_2_SeO_3_, respectively. Z50, Z51, Z52, Z53, and M54 represented mycelia grown for 5 weeks in the presence of 0, 0.1, 0.2, 0.3, or 0.4 mmol/L Na_2_SeO_3_, respectively. Z60, Z61, Z62, Z63, and Z64 represented mycelia grown for 6 weeks in the presence of 0, 0.1, 0.2, 0.3, or 0.4 mmol/L Na_2_SeO_3_, respectively. Z70, Z71, Z72, Z73, and Z74 represented mycelia grown for 7 weeks in the presence of 0, 0.1, 0.2, 0.3, or 0.4 mmol/L Na_2_SeO_3_, respectively. Z80, Z81, Z82, Z83, and Z84 represented mycelia grown for 8 weeks in the presence of 0, 0.1, 0.2, 0.3, or 0.4 mmol/L Na_2_SeO_3_, respectively

Principal component analysis (PCA) was used to determine the difference between treated samples at different time points (Fig. 2). The first principal component showed the greatest contribution (71.41%), whereas the second principal component contributed 14.37% of the total, indicating a clear distinction between samples cultured under different conditions (cultivation time).

**Figure 3:**
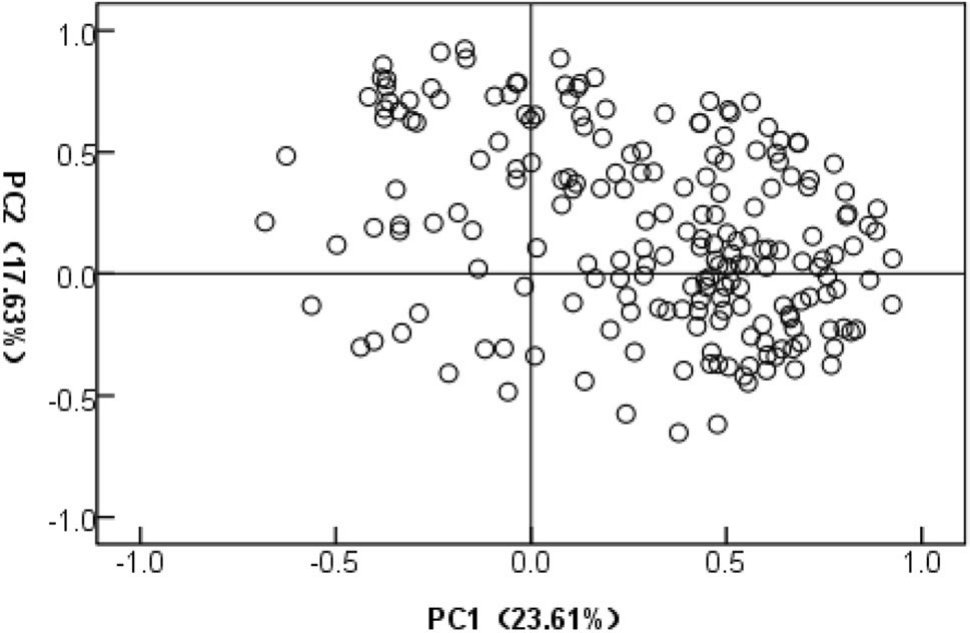
Principal component analysis score plot of first and second PCs from 203 metabolites in mycelia of *Epichloë* sp.

We carried out a principal component analysis to visualize the differences among these metabolites (Fig. 3). Three primary components were identified in an analysis of 203 metabolites, with the three components accounting for approximately 53.45% of the total variance. Metabolites contributing to the first component included lactamide, malonic acid, methylmalonic acid, 2-butyne-1,4-diol, ethanolamine, phosphate, succinic acid, picolinic acid, itaconic acid, malonamide, L-homoserine, 3-aminoisobutyric acid, and 6-phosphogluconic acid. Metabolites contributing to the second component included 5-dihydrocortisol, cholecalciferol, 4-androsten-11-β-ol-3,17-dione, galactinol, hydrocortisone, heptadecanoic acid, and palmitic acid.

### Changes in the Metabolite Profiles of Epichloë sp. from F. sinensis in Response to Se

**Figure 4:**
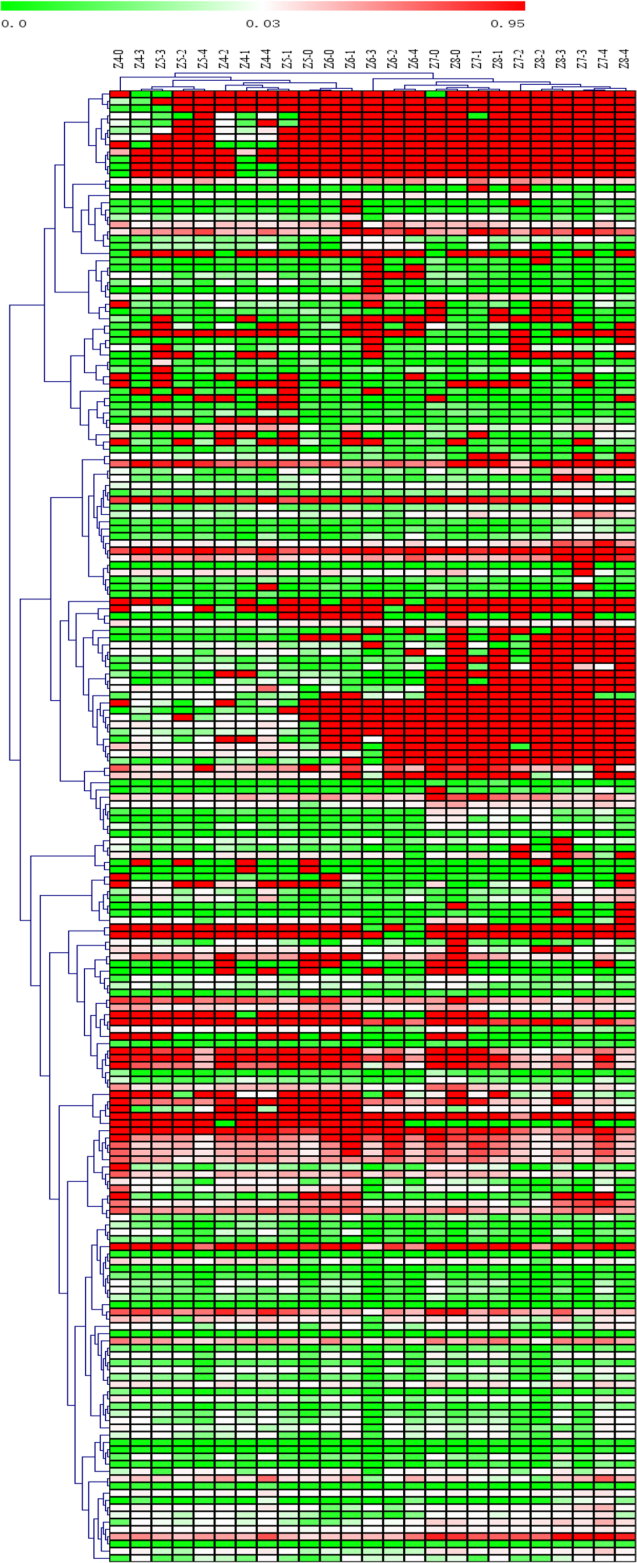
Heatmap and hierarchical cluster analysis for the 203 metabolites in *Epichloë* sp. mycelia. The dendogram for metabolite clustering was shown on the top of the heatmap and sample clustering was displayed on the right. Red and green blocks indicated a higher and lower metabolite levels (see scale bar). Z4-0, Z4-1, Z4-2, Z4-3, and Z4-4 represented mycelia grown for 4 weeks in the presence of 0, 0.1, 0.2, 0.3, or 0.4 mmol/L Se, respectively. Z5-0, Z5-1, Z5-2, Z5-3, and Z5-4 represented mycelia grown for 5 weeks in the presence of 0, 0.1, 0.2, 0.3, or 0.4 mmol/L Se, respectively. Z6-0, Z6-1, Z6-2, Z6-3, and Z6-4 represented mycelia grown for 6 weeks in the presence of 0, 0.1, 0.2, 0.3, or 0.4 mmol/L Se, respectively. Z7-0, Z7-1, Z7-2, Z7-3, and Z7-4 represented mycelia grown for 7 weeks in the presence of 0, 0.1, 0.2, 0.3, or 0.4 mmol/L Se, respectively. Z8-0, Z8-1, Z8-2, Z8-3, and Z8-4 represented mycelia grown for 8 weeks in the presence of 0, 0.1, 0.2, 0.3, or 0.4 mmol/L Se, respectively

The heatmap and hierarchical cluster analysis were used to identify the different metabolites between samples in response to Se concentrations in all metabolite profiles of 25 *Epichloë* sp. samples. According to hierarchical clustering analysis of the 203 identified metabolites, a clear separation between samples was observed, with 25 samples grouped into two categories: one category included 12 samples (both the control and treated samples between weeks 4 and 5, as well as samples with control and 0.1 mmol/L Na_2_SeO_3_ in the sixth week), and the other category included the remaining 13 samples (Fig. 4). Additionally, 203 metabolites were classified into two groups, each with 153 and 50 compounds, respectively.

Among the 203 identified metabolites (Table 2), 15 metabolites displayed differential accumulation in the 0.1 mmol/L Se group compared to the CK group (0.1 mmol/L vs. CK), seven of these significantly altered metabolites showed increased levels, while eight showed decreased levels in the 0.1 mmol/L Se treatment group. We observed an increase in 12 metabolites and a decrease in 34 metabolites in 0.2 mmol/L vs. CK. In 0.3 mmol/L vs. CK, 17 metabolites increased and 15 decreased, whereas 24 metabolites increased and 25 decreased in 0.4 mmol/L vs 0 mmol/L samples. These findings indicated that Na_2_SeO_3_ concentrations affected metabolite alterations. Parallel to the cultivation period, the number of metabolites was either upregulated or downregulated (Table 3). When comparing the Se treatment to the control group, the elevated metabolites in the fifth week were significantly higher than at other time points. In addition, when comparing the Se therapy to the control group, the lowered metabolites in the 5th week were much lower than the elevated metabolites. The samples exhibited metabolic complexity in terms of cultivation time and Se treatments.

**Table 2:** Number of increased or decreased metabolite levels among 203 identified metabolites in *Epichloë* sp. mycelia from different Se concentration and control groups

Change	Number of metabolites			
	0.1 mmol/L vs. CK	0.2 mmol/L vs. CK	0.3 mmol/L vs. CK	0.4 mmol/L vs. CK
Increase	7	12	17	24
Decrease	8	34	15	25

**Table 3:** Changes in metabolite profiles in *Epichloë* sp. mycelia between Se and control groups at weeks 4~8

Change in Se vs. CK	Number of metabolites				
	Week 4	Week 5	Week 6	Week 7	Week 8
Increase	48	67	31	18	20
Decrease	66	32	67	72	57

### Effects of Selenium on Amino Acid, Organic Acid of TCA, Fatty Acid, Carbohydrate, and Other Compounds

**Figure 5:**
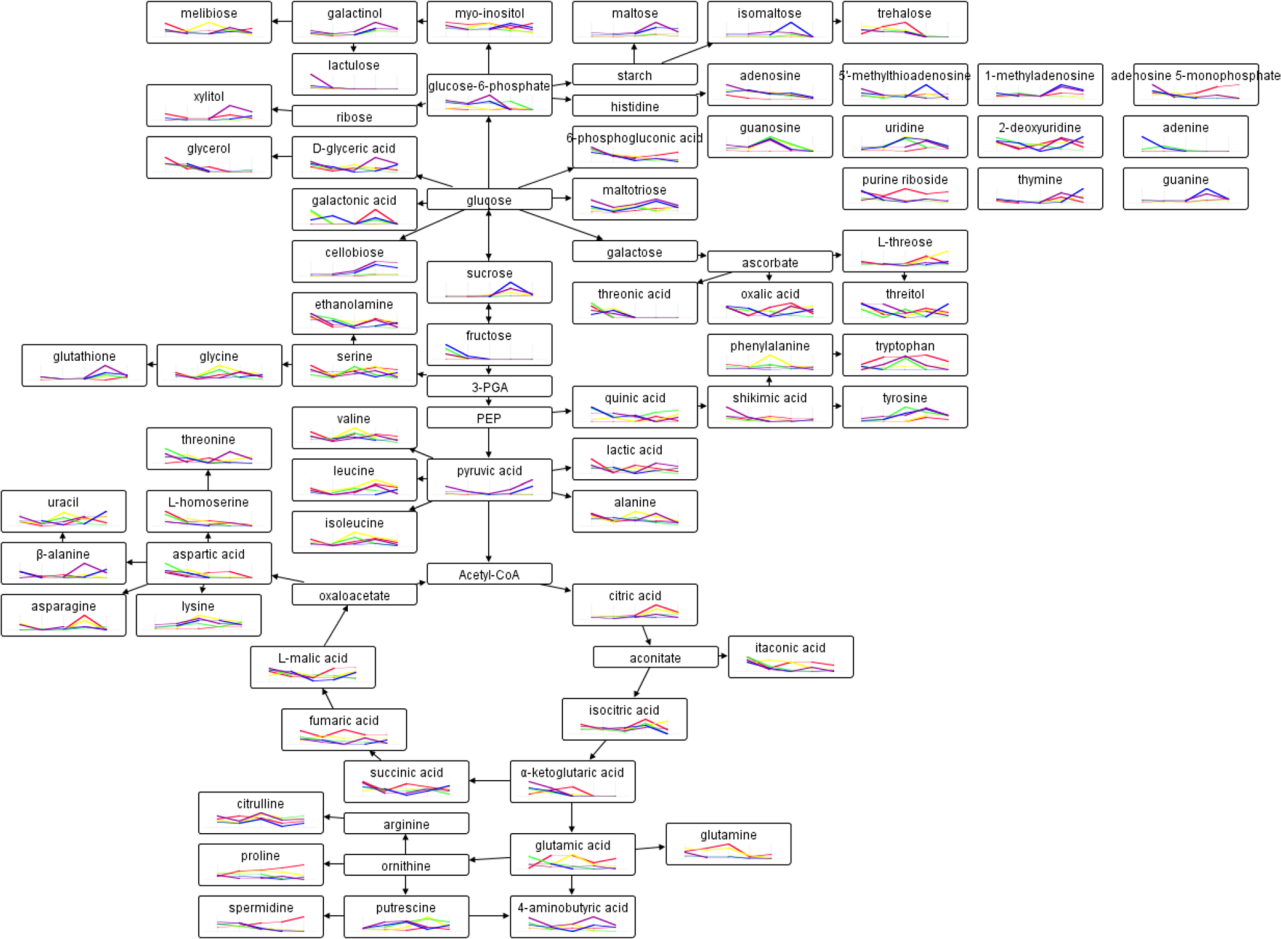
Map of measured primary metabolome of *Epichloë* sp. mycelia. Metabolic pathway map for red, yellow, green, blue, and purple colors represented selenium concentrations of 0, 0.1, 0.2, 0.3, or 0.4 mmol/L, respectively. 3-PGA, 3-phosphoglycerate; PEP, phosphoenolpyruvate

**Figure 6:**
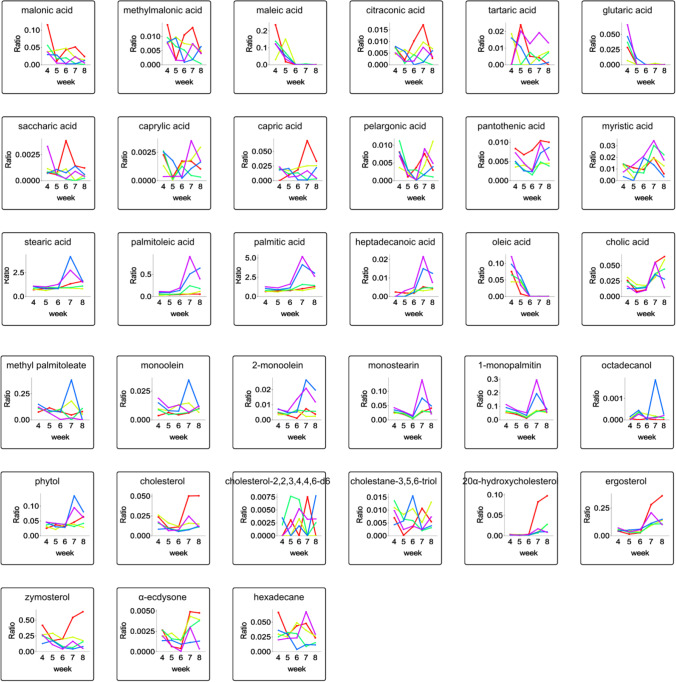
Time stamp of fatty acid, esters, and alcohol abundance in *Epichloë* sp. mycelia under each selenium concentration. Red, yellow, green, blue, and purple colors represented selenium concentrations of 0, 0.1, 0.2, 0.3, or 0.4 mmol/L, respectively

A biochemical pathway model for *Epichloë* sp. from *F. sinensis* was constructed (Fig. 5). Se treatment had a significant effect on 131 mycelial metabolites within the same culture time, and culture time had a significant (*P*<0.05) effect on 121 metabolites at each selenium concentration. Additionally, the abundances of maltose, adenosine, uridine, and 2-keto-isovaleric acid were higher under selenite conditions than under normal conditions (*P*<0.001). In comparison, selenite treatment resulted in a decrease in pantothenic acid (Fig. 6). Additionally, we observed higher glutathione levels in *Epichloë* sp. grown with selenite supplementation (0.1~0.4 mmol/L) in the 4th, 5th, and 7th weeks, respectively.

### Effect of Selenite on Amino Acid

Thirteen amino acids’ accumulation was enhanced to varying degrees following selenite therapy, whereas six amino acids appeared to have no positive association with selenite treatment. The highest levels of alanine, valine, isoleucine, glycine, glutamic acid, phenylalanine, and lysine were obtained in the mycelia after the addition of 0.1 mmol/L Na_2_SeO_3_ in the 6th week, and leucine after the addition of 0.1 mmol/L Na_2_SeO_3_ in the 7 weeks of cultivation. Threonine and aspartic acid concentrations were highest in the presence of 0.2 mmol/L Na_2_SeO_3_ in week 4, and tyrosine concentrations were highest in the mycelia after the addition of 0.2 mmol/L Na_2_SeO_3_ in week 6. Additionally, citrulline and β-alanine achieved maximum levels at a concentration of 0.4 mmol/L Na_2_SeO_3_ in the sixth and seventh weeks, respectively, whereas serine, homeserine, glutamine, asparagine, tryptophan, and proline concentrations reached their maximum levels in the absence of Na_2_SeO_3_ in the 4th, 5th, 6th, 7th, and 8th weeks, respectively.

The comparison of changes in several amino acids from *Epichloë* sp. mycelia between the Se and control groups is shown in Figs. 4 and 5. When compared to the control samples, proline decreased with Se treatment across time points, whereas lysine increased from 4 to 6 weeks, and then increased in the 8th week. In the fourth week, three compounds containing 19 amino acids exhibited an increase in Se while eight compounds exhibited a decrease in Se when compared to the control (Se vs. CK). Between Se and control at week 5, three amino acids (alanine, glycine, and lysine) were upregulated, while six amino acids were downregulated. In the 6th week, only lysine concentration was found to be higher in selenite samples than in the control samples, whereas six amino acids were found to be lower in selenite samples than in control samples. Only tyrosine increased while six amino acids decreased in Se vs. CK after the seventh week of culture. Three amino acids accumulated over the final week of cultivation, while seven amino acids in mycelia were suppressed by adding selenite.

### Effect of Selenite on Organic Acids of the Tricarboxylic Acid Cycle (TCA)

The majority of the organic acids in TCA that changed were decreased over time in mycelia treated with 0.1~0.4 mmol/L Se at different time points. When compared to the control, citric acid and fumaric acid were increased by 0.1 mmol/L Na_2_SeO_3_ between 4~5 weeks, while succinic acid was increased by 0.1~0.3 mmol/L Na_2_SeO_3_ in weeks 5 and 8. A similar increase in malic acid levels was observed in the 4th week by 0.2~0.4 mmol/L Se, in the 5th week by all selenite treatment, and by 0.1, 0.2, and 0.4 mmol/L selenite treatment in the 6th week. Even more significantly, when compared with the control, α-ketoglutaric acid levels were elevated across all selenite concentrations in the fourth week as well as by 0.4 mmol/L Na_2_SeO_3_ in the fifth week.

### Effect of Selenite on Fatty Acid

The fatty acid profile produced by a microorganism depends on the microorganism species, culture conditions, and duration of culture. *Epichloë* sp. was found to have a significant amount of stearic acid (C18:0), with the highest proportion detected in mycelia treated with 0.3 mmol/L selenite during 7 weeks of cultivation (Fig. 6). Furthermore, increased levels of stearic acid, palmitoleic acid, and palmitic acid were found in the mycelia supplemented with a specific amount of selenium from 4 to 8 weeks when compared to the levels found in the control mycelia. In the first 4~5 weeks of cultivation, high selenite concentrations increased the levels of capric acid, saccharic acid, and oleic acid; however, selenite concentrations decreased the levels of these acids between the 6th and 8th weeks of cultivation. During the 4th and 8th weeks of cultivation, the changes in pelargonic acid and citraconic acid were elevated, but the changes in citraconic acid were downregulated from the 5th to 7th weeks of cultivation in the presence of selenite treatments. During the period of cultivation, selenite at 0.1~0.4 mmol/L reduced the production of pantothenic acid.

Myristic acid and tartaric acid levels increased in response to Se concentrations of 0.1, 0.2, and 0.3 mmol/L in the 4th week and between the 6th and 8th weeks. Control mycelia were characterized by increasing amounts of heptadecanoic acid and malonic acid at this first-time point, and selenite reduced the production of heptadecanoic acid in mycelia from the 5th to the 8th week. Furthermore, mycelia enriched with 0.1 or 0.4 mmol/L selenite demonstrated that the concentration of hexadecane increased during the last week of cultivation when compared to mycelia grown without the addition of this element.

### Effects of Selenite on Carbohydrate and Other Compounds

When compared to the control, maltose concentrations increased across all selenite concentrations and time points. Sucrose levels increased in the 5th, 7th, and 8th weeks following cultivation when compared to the control. As opposed to the control group, fructose levels of mycelia were slightly increased by 0.2~0.4 mmol/L Na_2_SeO_3_ between the 4th and 5th weeks. Cellobiose levels were significantly reduced by 0.1 mmol/L selenium in the 4th week and by 0.4 mmol/L selenite in the 7th week compared with the control. Trehalose levels increased at this first-time point across all selenite treatments, as well as at week 7 in the presence of 0.2 mmol/L Na_2_SeO_3_. When compared to the control, the isomaltose levels of mycelia were slightly decreased by 0.1 mmol/L Na_2_SeO_3_ in the 6th week. During the fourth week of cultivation, all selenite treatments significantly decreased melibiose levels, whereas the 0.1 mmol/L Na_2_SeO_3_ in the culture medium significantly increased melibiose levels in the 6th week of cultivation (*P*<0.05).

During the 6th week of cultivation in experimental media supplemented with 0.1 mmol/L selenium concentration as well as the 7th week of cultivation in both the 0.3 and 0.4 mmol/L Se culture media, an increase in myo-inositol was detected. Between weeks 5 and 6, galactinol concentrations increased in the presence of all Se concentrations, as well as in weeks 4, 7, and 8 of cultivation in the presence of 0.3 or 0.4 mmol/L Na_2_SeO_3_. *Epichloë* sp. mycelia with 0.4 mmol/L or 0.3 mmol/L Se had significantly higher putrescine concentrations than the control in weeks 5 of cultivation (*P*<0.05). Mycelia with 0.1~0.2 mmol/L Se had significantly higher putrescine concentrations than those in the control group at the 7th week (*P*<0.05). Selenium at 0.3 and 0.4 mmol/L concentrations significantly increased spermidine production in mycelia in the 5th week (*P*<0.05). Within 4~8 weeks of cultivation, 4-aminobutyric acid levels in mycelia under 2~4 different selenite treatments were higher than those in the control mycelia.

While selenite supplementation decreased the concentration of zymosterol, higher zymosterol levels were seen in *Epichloë* sp. mycelia treated with 0.1 and 0.2 mmol/L selenium in the 5th week, compared to control mycelia (Fig. 6). Between the 4th and 6th weeks of culture in PDA enriched with selenite, the ergosterol concentration increased and then decreased between the 7th and 8th weeks. In the fifth week of cultivation, mycelia treated with 0.1 mmol/L selenite had increased cholesterol, 20α-hydroxycholesterol, and cholestane-3,5,6-triol concentrations.

### Marked Metabolites Changed Under Selenium Condition

**Figure 7:**
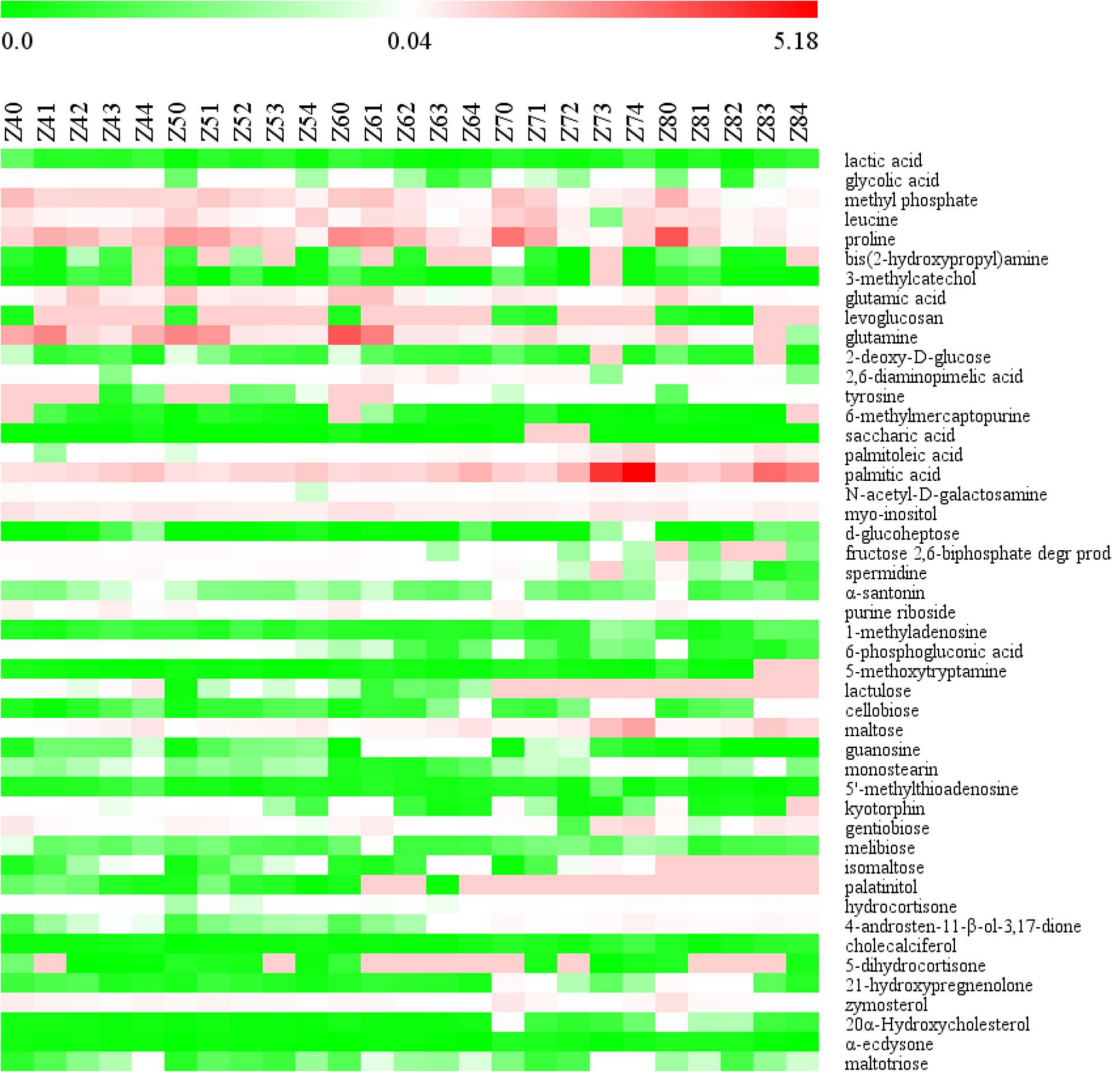
Time stamp of the abundance of marked metabolites in *Epichloë* sp. under selenium concentrations among a given culture time. Red and green blocks indicated a higher and lower metabolite levels (see scale bar). Z40, Z41, Z42, Z43, and Z44 represented mycelia grown for 4 weeks in the presence of 0, 0.1, 0.2, 0.3, or 0.4 mmol/L Na_2_SeO_3_, respectively. Z50, Z51, Z52, Z53, and M54 represented mycelia grown for 5 weeks in the presence of 0, 0.1, 0.2, 0.3, or 0.4 mmol/L Na_2_SeO_3_, respectively. Z60, Z61, Z62, Z63, and Z64 represented mycelia grown for 6 weeks in the presence of 0, 0.1, 0.2, 0.3, or 0.4 mmol/L Na_2_SeO_3_, respectively. Z70, Z71, Z72, Z73, and Z74 represented mycelia grown for 7 weeks in the presence of 0, 0.1, 0.2, 0.3, or 0.4 mmol/L Na_2_SeO_3_, respectively. Z80, Z81, Z82, Z83, and Z84 represented mycelia grown for 8 weeks in the presence of 0, 0.1, 0.2, 0.3, or 0.4 mmol/L Na_2_SeO_3_, respectively

**Figure 8:**
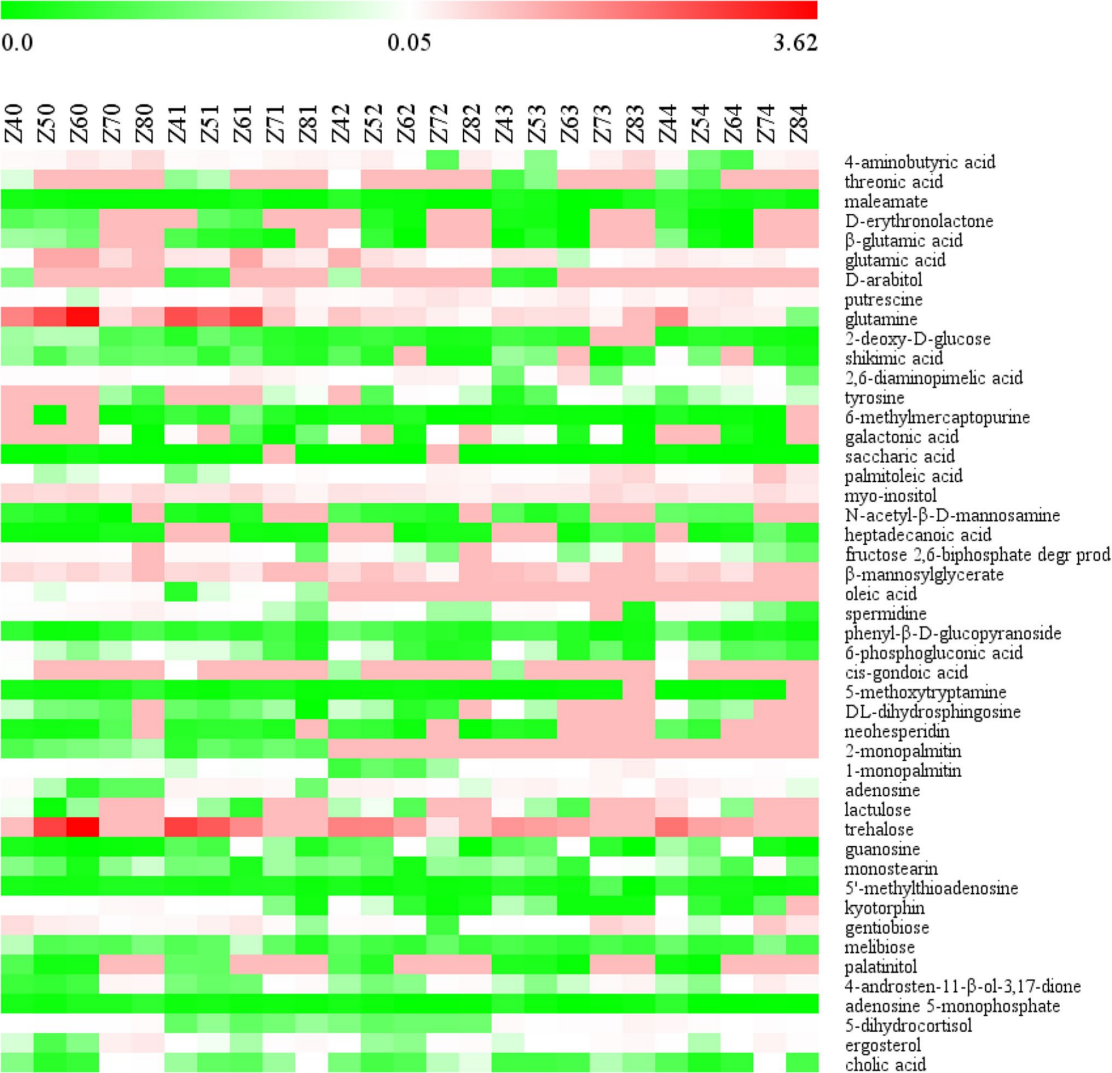
Time stamp of the abundance of marked metabolites in *Epichloë* sp. under each selenium concentration. Red and green blocks indicated a higher and lower metabolite levels (see scale bar). Z40, Z50, Z60, Z70, and Z80 represented mycelia grown in absense of Se for 4, 5, 6, 7, and 8 weeks, respectively. Z41, Z51, Z61, Z71, and Z81 represented mycelia grown in the presence of 0.1 mmol/L Se between 4~8 weeks. Z42, Z52, Z62, Z72, and Z82 represented mycelia grown in the presence of 0.2 mmol/L Se during weeks 4~8. Z43, Z53, Z63, Z73, and Z83 represented mycelia grown in the presence of 0.3 mmol/L Se from the 4th to 8th weeks. Z44, Z54, Z64, Z74, and Z84 represented mycelia grown in the presence of 0.4 mmol/L Se between weeks 4 and 8

The effects of Se treatment and treatment duration on the metabolite characteristics of *Epichlo* sp. mycelia were highly significant (*P*<0.001). The heatmap visualization demonstrated distinct trends in the changes in marked metabolites for each time point of exposure to Se concentrations and each Se concentration (Figs. 7 and 8). In weeks 4, 5, 6, 7, and 8, respectively, 15, 13, 36, 20, and 8 distinct metabolites were detected in mycelia (Fig. 7). At Se values of 0, 0.1, 0.2, 0.3, and 0.4 mmol/L, respectively, 6, 11, 29, 8, and 17 marked metabolites were found in mycelia during the culture process (Fig. 8).

The 47 marked metabolites showed significantly distinct variations for *Epichloë* sp. from *F. sinensis* in response to selenite concentrations at a specific culture time (Fig. 7) (*P*<0.001). When selenite-treated samples were compared to control samples at various time intervals, a decreasing number of marked metabolites were upregulated (Table 4). We compared 47 marked metabolites from *Epichloë* sp. grown in selenite and non-selenite conditions. Eleven metabolites were found to increase while 13 metabolites were found to decrease in Se compared to control in the 4th week. In selenite conditions, 15 metabolites increased and 13 metabolites decreased in the 5th week, 10 metabolites increased, and 19 metabolites decreased in the 6th week. Of note, the common upregulation of five metabolites (maltose, guanosine, 4-androsten-11-β-ol-3,17-dione, cholecalciferol, maltotriose) occurred between weeks 4 and 6. The 15 most common downregulated metabolites were detected in both weeks 7 and 8 under selenite conditions.

**Table 4:** Changes in levels of marked metabolites in *Epichloë* sp. mycelia from different Se and control groups at weeks 4~8

Change in Se vs. CK	Number of marked metabolites				
	Week 4	Week 5	Week 6	Week 7	Week 8
Increase	11	15	10	7	4
Decrease	13	13	19	19	25

For 47 mycelia metabolites, a highly significant effect of culture time was observed (Fig. 8) (*P*<0.001). As compared with the 4-week control mycelia, 4-aminobutyric acid, glutamic acid, spermidine, and 5-dihydrocortisol were upregulated, while nineteen compounds including threonic acid, spermidine, shikimic acid, monopalmitin, palatinitol, gentiobiose, melibiose, adenosine, and guanosine were downregulated between the 5th and 8th weeks. Between weeks 5 and 8, when 0.1 mmol/L Na_2_SeO_3_ was used, palmitoleic acid, oleic acid, and monopalmitin abundances were significantly greater than those before treatment (*P*<0.001). Additionally, four metabolites for 6-phosphogluconic acid, cis-gondoic acid, trehalose, and palatinitol under selenite treatments, and maleamate, glutamic acid, glutamine, shikimic acid, fructose 2,6-biphosphate degr prod, DL-dihydrosphingosine, neohesperidin, and adenosine 5-monophosphate under 0.2, 0.3, and 0.4 mmol/L selenite treatment dramatically decreased from the 5th to 8th weeks when compared with the 4-week samples added the same selenium concentration (*P*<0.001).

## Discussion

Selenium alters the phenotypic, physiological, and biochemical changes in fungi. In this study, we investigated the changes in growth and metabolites in *Epichloë* sp. exposed to different concentrations of sodium selenite to elucidate the mechanism of fungal survival under selenite conditions. Selenite treatment significantly altered amino acids, carbohydrates, organic acids, nucleotides, and their metabolites.

Selenium is widely implicated in fungal growth [12, 30, 31]. Diverse fungal species may exhibit varying levels of selenium tolerance. Consistent with this, our data demonstrated that when selenite concentrations were increased, the colony diameter of *Epichloë* sp. decreased, although the inhibitory effect was diminished after prolonged selenium stress (Table 1, Fig. 1). Thus, *Epichloë* sp. is likely to have continually absorbed and then depleted the medium’s Se concentration. According to its function, the application of microbial agents such as *Epichloë* sp. would help remediate soils polluted with selenium. Additionally, we noted that high selenite concentrations resulted in the red coloring of medium or mycelia (Fig. 1). Previous research has produced comparable findings. When grown on high Se dosage media, *Penicillium expansum*, *Aureobasidium pullulans*, *Mortierella humilis*, and *Phoma glomerata* all exhibit a similar phenotype [30, 32]. This could be because when certain fungi reduce elemental selenium from inorganic sodium selenite [4, 12], the hyphal matrix provides sites for the resulting Se nanoparticles [32]. Selenium is also incorporated into other biological macromolecules, including proteins [33]. The color change of the colony is closely related to a variety of extracellular compounds including sugars, proteins, organic acids, and alcohols [32, 34–36].

Elevated levels of some amino acids are essential in microbial responses to selenium [11]. Enrichment of Se has been associated with some amino acid levels in this study. After Se supplementation, there were variations in the levels of alanine, valine, isoleucine, glycine, glutamic acid, phenylalanine, lysine, leucine, threonine, aspartic acid, tyrosine, citrulline, and β-alanine. On the contrary, serine, homeserine, glutamine, asparagine, tryptophan, and proline concentrations peaked without the addition of selenite. In *Cordyceps militaris*, selenium at 60 mg/L suppressed serine and tyrosine levels [6]. Kieliszek et al. [11] found that proline, glutamine, alanine, arginine, and methionine levels varied between *Candida utilis* and *Saccharomyces cerevisiae* enriched with 20 mg Se^4+^/L culture media, compared to the control group. Consistent with this finding, the concentrations of individual amino acids are subject to the presence of selenium in culture conditions [37]. Therefore, it is likely that selenium helps maintain the reductive environment for the catalytic efficiencies of numerous enzymes, and simulates the biosynthesis of various amino acids. Glutathione refers to a combination of glycine, glutamic acid, and cysteine, and is involved in cellular processes of detoxification. In addition, we detected elevated levels of glutathione at weeks 4, 5, and 7 in *Epichloë* sp. grown under selenite supplementation (0.1~0.4 mmol/L). Total glutathione levels in the biomass of *C. utilis* and *S. cerevisiae* yeasts were found to increase with increasing selenium levels [8]. Glutathione, in combination with lipids and proteins, forms part of hyphal cell walls. Elemental selenium is absorbed by the mycelia. Rao et al. [38] reported that exposure to selenite suppresses glutathione levels. The absorption of Sodium selenite in microorganisms is mediated by glutathione, which non-enzymatically reacts with selenium to form selenodiglutathione (GS-Se-SG) [39, 40]. Selenodiglutathione is further metabolized to elemental Se by glutaredoxin systems. Our data showed that selenium increased 2-keto-isovaleric acid levels, which may indirectly explain why mycelia had higher valine levels under selenium conditions, compared to the control. Amino acids are necessary for mycelial growth and reproduction, since they are involved in nucleotide and lipid biosynthesis.

Polyamines are formed via decarboxylation processes of amino acids such as ornithine and arginine. They are involved in maintenance of ionic balance in cells, thereby enhancing microbial tolerance. In addition, they are involved in responses to diverse environmental stressors. In various organisms, abiotic stress was shown to modulate the accumulation of polyamines and related gene expressions. In this study, putrescine was significantly increased in most selenium treatment groups, implying that putrescine may play an important role in microbial adaptation to various kinds of stress [41, 42]. Additionally, spermidine, another polyamine compound that has been associated with increasing cell lifespan and suppression of oxidative stress, was elevated at weeks 4 and 8 in all selenite treatment groups as well as in the 5th week in mycelia treated with 0.3 mmol/L and 0.4 mmol/L Na_2_SeO_3_, relative to the control. The protein encoded by spermidine synthase was highly abundant in one Se yeast product [43]. 4-aminobutytic acid, one of the most important derivatives of polyamine oxidase, plays a metabolic role in Krebs cycle. We established that in different selenite treatment groups, 4-aminobutytic acid levels were increased at weeks 4 to 8 by factors of 2~4. In tandem with our findings, Su et al. [44] reported elevated GABA levels in solid-state fermentation of *Monascus purpureus* when the basic medium was supplemented with both NaNO_3_ and MnSO_4_·H_2_O. Incubation period, monosodium glutamate, and pH level of the culture media exert positive effects on GABA levels in *M. sanguineus* [45].

An association between selenium and fatty acids has been reported [46]. Kieliszek et al. [11] found that the main fatty acid in yeast was oleic acid (C18:1), and *C. utilis* without added selenium exhibited a high abundance of oleic acid (C18:1), stearic acid (C18:0), palmitoleic acid (C16:1), and myristic acid (C14:0), compared to the culture with selenium supplementation. However, selenium supplementation enhanced the levels of oleic acid, palmitoleic acid, and myristic acid in *S. cerevisiae*. Moreover, yeasts treated with selenium produced large amounts of linoleic acid and linolenic acid [46]. Under 90 μg/mL Se treatment, Guan et al. [47] reported elevated levels of arachidonic acid in *Diasporangium jonesianum*; however, the levels of myristic acid (14:0), hexadecenoic acids (16:1), and octadecenoic (18:1) were suppressed. Our findings suggest that at appropriate concentrations, selenium increased stearic acid, palmitoleic acid, and palmitic acid levels in the mycelia across the different time points, compared to those of the control mycelia. In addition, there were significant differences in fatty acid profiles between the different concentrations of selenium and culture time. The deviations in fatty acid levels might be due to variations in microbial species. Therefore, selenium induces lipid peroxidation, which leads to the loss of cytoplasmic membrane integrity.

Glutathione affects the activities of carbohydrate metabolism-associated enzymes. Some carbohydrates are involved in maintenance of structural integrity of cells under several adverse environmental conditions. Compared to the control group, at 20 mg/L Se, carbohydrates from mycelia were significant in *P. expansum* [30]. The addition of 0.1 mmol/L sodium selenite decreased trehalose contents in *S. cerevisiae* [48]. In this study, it was determined that the addition of selenium to the culture medium increased the concentrations of maltose, isomaltose, melibiose, trehalose, sucrose, and fructose at specific time points. Specifically, selenium significantly increased mycelial maltose and melibiose levels (*P*<0.001) at weeks 4~6. These findings suggest that Se plays a key role in cell sugar uptake and transport. Gómez-Gómez et al. [14] reported that selenite upregulated the levels of exopolysaccharide production-associated proteins while downregulating sugar degradation-related enzymes, such as maltose phosphorylase and beta-galactosidase in *Lactobacillus reuteri* CRL1101. However, different species have evolved their metabolome for specific metabolites. For instance, treatment of fungi with 1 mmol/L Na_2_SeO_3_ was associated with varying levels of exopolysaccharides in *Trichoderma harzianum*, *Aureobasidium pullulans*, *Mortierella humilis*, and *Phoma glomerata* [32].

Most purines and pyrimidines are present in cells as nucleotides, and they are involved in the biosynthesis of genetic information carriers (DNA and RNA) or energy suppliers (ATP and GTP) [49]. Adenosine levels in *C. militaris* were found to increase with increasing Se concentrations [6]. In this study, adenosine levels in the mycelia of *Epichloë* sp. grown with the selenium supplement were increased. However, purine riboside levels in mycelia exposed to Se at weeks 6 and 8 were significantly low (*P*<0.001). Hu et al. [50] reported high adenosine levels in *C.* *militaris* enriched with selenium, which inhibited or initiated purine and pyrimidine metabolism. These results suggest that Se affects cellular DNA and RNA synthesis during this period, thereby interfering with mycelial growth and progagation.

Ergosterol is a vitamin D precursor and plays a pivotal role in membrane fluidity. Supplementation of Se to *Pleurotus ostreatus* and *Ganoderma lucidum* growth media resulted in reduced ergosterol levels in fruiting bodies, compared to the control group [51]. Moreover, relative to the control group, the Se supplementation group exhibited the highest ergosterol levels between weeks 4 and 6, which then reduced after week 7. To the best of our knowledge, this is the first study to report the effects of sodium selenite on *Epichloë* sp. from *F*. *sinensis*.

## Conclusion

We used metabolomic data to evaluate differences in metabolic responses of *Epichloë* sp. to Se. The number of metabolites in 0.4 mmol/L Se vs. CK treatment groups was higher than in the other Se treatment vs. CK treatment groups, and these metabolites were also elevated at week 5 of cultivation, relative to other time points. The levels of maltose, adenosine, uridine, and 2-keto-isovaleric acid, which are involved in polysaccharide, nucleic acid, and amino acid biosynthesis, were associated with responses to selenium. The glutathione metabolism pathway has a significant role in selenium metabolism. Studies on gene expressions and morpho-physiological responses should be performed to determine selenium tolerance-associated mechanisms.

## Supplementary Information

Below is the link to the electronic supplementary material.

Supplementary file1 (PDF 819 KB)

## Data Availability

The raw chromatogram is in the supplemental figure.
