# Supplementary material for: Effect of Sodium Selenite on the Metabolite Profile of Epichloë sp. Mycelia from Festuca sinensis in Solid Culture
Source: Biol Trace Elem Res. 2022 Jan 1;200(11):4865–79. doi: 10.1007/s12011-021-03054-w (PMC9492591; doi:10.1007/s12011-021-03054-w)
Supplement: Supplementary file 1 — Supplementary file1 (PDF 819 KB) [file 12011_2021_3054_MOESM1_ESM.pdf]

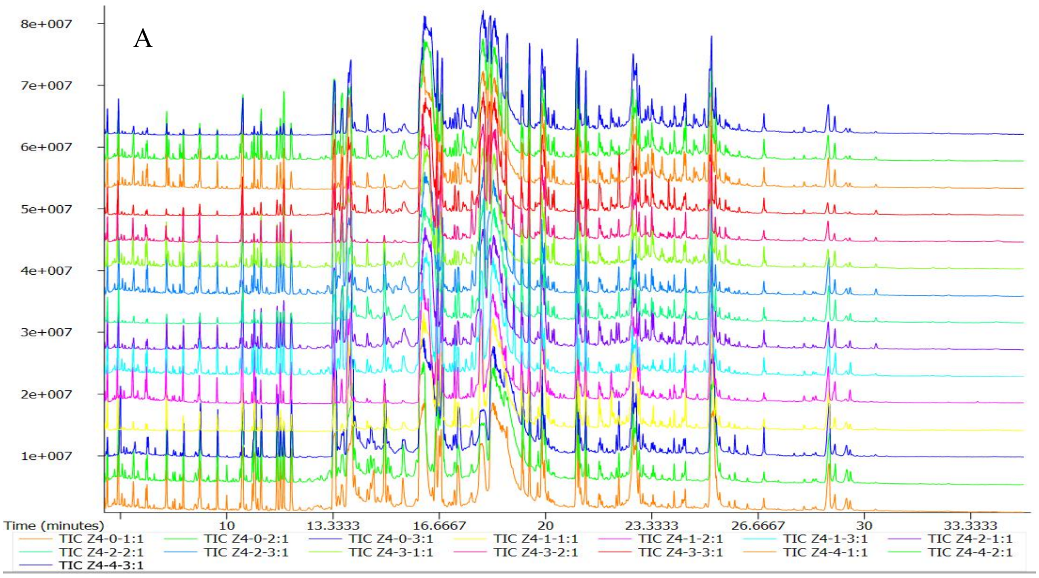
A
8e+007
7e+007
6e+007
5e+007
4e+007
3e+007
2e+007
1e+007
Time (minutes)
10
13.3333
16.6667
20
23.3333
26.6667
30
33.3333
TIC Z4-0-1:1
TIC Z4-0-2:1
TIC Z4-0-3:1
TIC Z4-1-1:1
TIC Z4-1-2:1
TIC Z4-1-3:1
TIC Z4-2-1:1
TIC Z4-2-2:1
TIC Z4-2-3:1
TIC Z4-3-1:1
TIC Z4-3-2:1
TIC Z4-3-3:1
TIC Z4-4-1:1
TIC Z4-4-2:1
TIC Z4-4-3:1

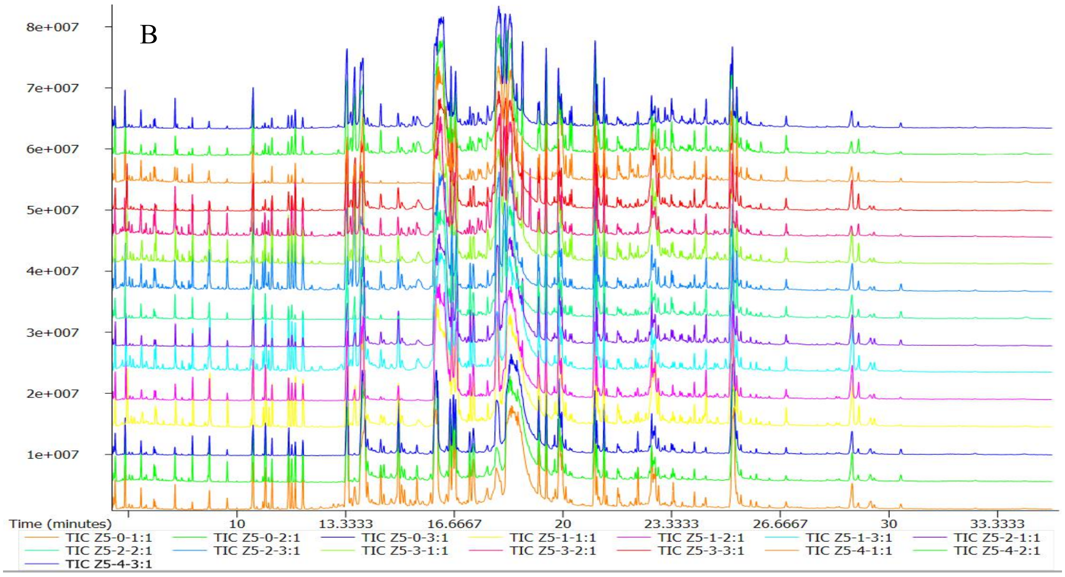
B
8e+007
7e+007
6e+007
5e+007
4e+007
3e+007
2e+007
1e+007
Time (minutes)
10
13.3333
16.6667
20
23.3333
26.6667
30
33.3333
TIC Z5-0-1:1
TIC Z5-0-2:1
TIC Z5-0-3:1
TIC Z5-1-1:1
TIC Z5-1-2:1
TIC Z5-1-3:1
TIC Z5-2-1:1
TIC Z5-2-2:1
TIC Z5-2-3:1
TIC Z5-3-1:1
TIC Z5-3-2:1
TIC Z5-3-3:1
TIC Z5-4-1:1
TIC Z5-4-2:1
TIC Z5-4-3:1

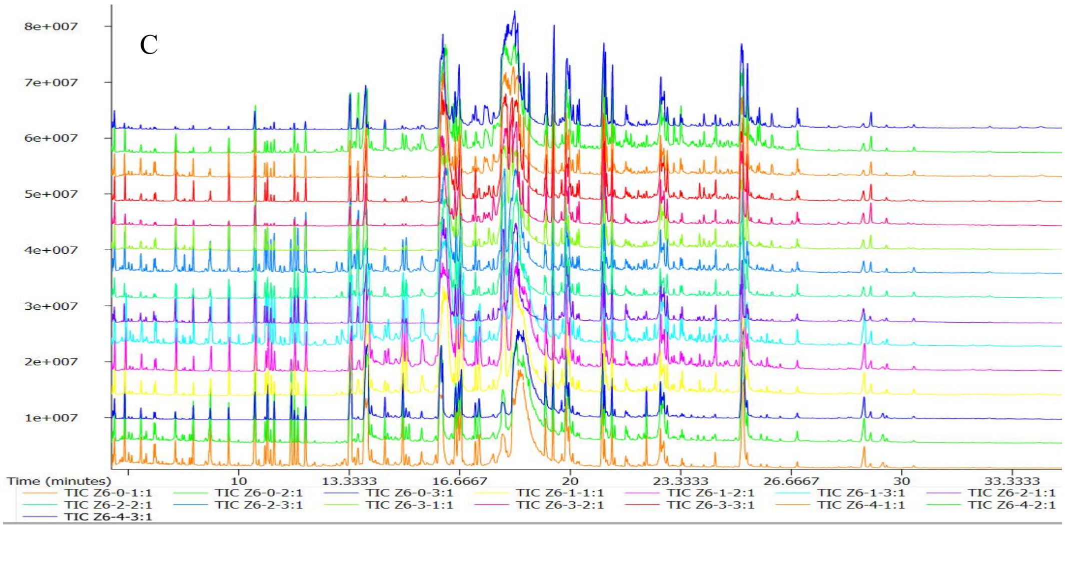
C
8e+007
7e+007
6e+007
5e+007
4e+007
3e+007
2e+007
1e+007
Time (minutes)
10
13.3333
16.6667
20
23.3333
26.6667
30
33.3333
TIC Z6-0-1:1
TIC Z6-0-2:1
TIC Z6-0-3:1
TIC Z6-1-1:1
TIC Z6-1-2:1
TIC Z6-1-3:1
TIC Z6-2-1:1
TIC Z6-2-2:1
TIC Z6-2-3:1
TIC Z6-3-1:1
TIC Z6-3-2:1
TIC Z6-3-3:1
TIC Z6-4-1:1
TIC Z6-4-2:1
TIC Z6-4-3:1

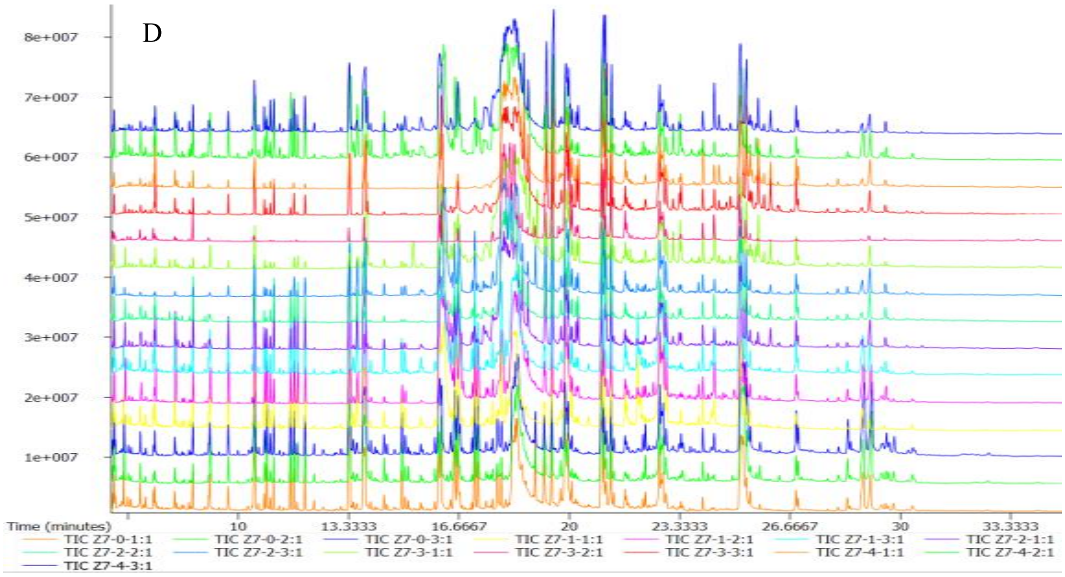

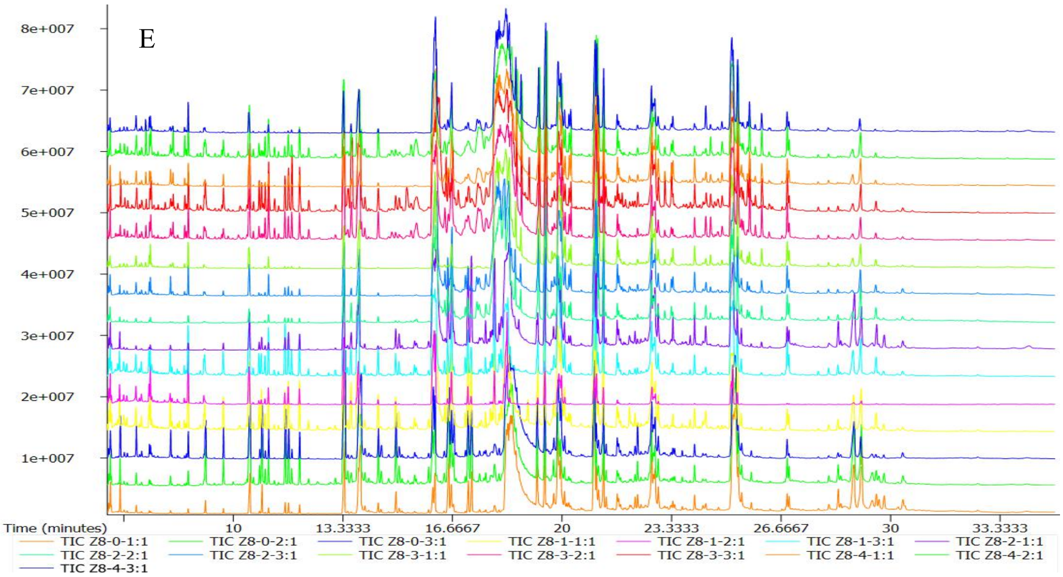

Figure S1 Total ion chromatogram of samples. Z4-0, Z4-1, Z4-2, Z4-3, and Z4-4 represented the treated 4 week samples for 0, 0.1, 0.2, 0.3, and 0.4 mmol/L of Se concentrations, respectively. Z5-0, Z5-1, Z5-2, Z5-3, and Z5-4 represented the treated 5 week samples for 0, 0.1, 0.2, 0.3, and 0.4 mmol/L of Se concentrations, respectively. Z6-0, Z6-1, Z6-2, Z6-3, and Z6-4 represented the treated 6 week samples for 0, 0.1, 0.2, 0.3, and 0.4 mmol/L of Se concentrations, respectively. Z7-0, Z7-1, Z7-2, Z7-3, and Z7-4 represented the treated 7 week samples for 0, 0.1, 0.2, 0.3, and 0.4 mmol/L of Se concentrations, respectively. Z8-0, Z8-1, Z8-2, Z8-3, and Z8-4 represented the treated 8 week samples for 0, 0.1, 0.2, 0.3, and 0.4 mmol/L of Se concentrations, respectively. -1, -2 and -3 represented the repetition number of sample.
